# Benefit, risk and cost of new oral anticoagulants and warfarin in atrial fibrillation; A multicriteria decision analysis

**DOI:** 10.1371/journal.pone.0196361

**Published:** 2018-05-03

**Authors:** Jose Mendoza-Sanchez, Federico Silva, Lady Rangel, Linda Jaramillo, Leidy Mendoza, Jenny Garzon, Andrea Quiroga

**Affiliations:** 1 Grupo de Ciencias Neurovasculares, Instituto Neurológico, Hospital Internacional, Fundación Cardiovascular de Colombia, Floridablanca, Santander, Colombia; 2 Universidad Industrial de Santander, Bucaramanga, Santander, Colombia; Universita degli Studi di Napoli Federico II, ITALY

## Abstract

**Introduction:**

Warfarin and new oral anticoagulants are effective in reducing stroke in atrial fibrillation; however, the benefits and risks rates in clinical trials show heterogeneity for each anticoagulant, and is unknown the cost influence on a model considering most of the treatment consequences. We designed a benefit-risk and cost assessment of oral anticoagulants.

**Design:**

We followed the roadmap proposed by IMI-PROTECT and the considerations of emerged good practice to perform Multi-Criteria Decision Analysis (MCDA). The roadmap defines the following steps: (1) planning, (2) evidence gathering and data preparation, (3) analyses, (4) explorations, and (5) conclusions. We defined two reference points (0–100) to allocate numerical values for scores and weights, and used an analogue numeric scale to assess physicians’ preferences.

As benefits of the anticoagulant therapy, we included reductions in stroke and all-cause mortality; intracranial haemorrhage, gastrointestinal haemorrhage, minor bleeding and myocardial infarction were considered risks. We also made an estimation of the annual drug cost per person.

**Main results:**

The scores were: Apixaban 33, Dabigatrán 25, warfarin 18 and Rivaroxaban 14 this score reveals the most preferred up to the less preferred option, considering the benefit-risk ratio and drug costs altogether. The relative model weights were: 51.1% for risks, 40.4% for benefits and 8.5% for cost. The sensitivity analysis confirms the model robustness.

**Conclusions:**

From this analysis, apixaban should be considered as the preferred anticoagulant option -due to a better benefit-risk balance and a minor cost influence- followed by dabigatran, warfarin and rivaroxaban.

## Introduction

Warfarin and new oral anticoagulants are effective in reducing stroke risk in atrial fibrillation (AF). However, oral anticoagulation drugs also have risks as: major bleeding (2% - 5% annual), fatal bleeding (0.5% - 1%) and intracranial haemorrhage (0.2% - 0.4% annual);Other sources of bleeding such as gastrointestinal haemorrhage, have been described with warfarin use [1]. New Oral Anticoagulants (NOACs) -apixaban, dabigatran and rivaroxaban-have showed heterogeneous results: Apixaban reduces risk of stroke without increasing risk of major bleeding or intracranial haemorrhage; dabigatran 150mg reduces risk of stroke with similar bleeding risk, but slightly increases gastrointestinal bleeding and myocardial infarction risk; rivaroxaban may be as effective as warfarin in preventing stroke or systemic embolism[2] [3] [4] [5]. In this context, the election of the most suitable anticoagulant becomes a task.

Doctors elect drugs based not only on their benefits and security profile but also on their costs, especially when patients have to pay because it impacts on drug outcomes [6] [7]. Cost seems to be the most important factor decision to prescribe dabigatran (cost 25%, renal function 21% and CHADS2 score 18%) and the second more often considered in patients with warfarin (unstable international ratio 37% and cost 19%) [8]. Thus, drugs evaluations with a holistic view (benefit-risk-cost) are useful to elect a drug. For assessments on which multiple points are considered, the Multicriteria Decision Analysis (MCDA) allows performing these evaluations. It provides a carefully balancing of benefits and harms as a numeric value; furthermore, it is widely used by pharmaceutical companies, regulators and academics to perform benefits-risk assessments (BRA) [9] [10].

Two MCDA for oral anticoagulants have been published, both show dabigatran 150mg as the preferred option [11] [12], one of them includes costs as a criterion, and both differ on criteria election as well as scores, criteria weighted and others. The cost inclusion in this model was with the aim of evaluate events´ cost from a payer perspective. But doctors´ decisions tend to integrate multiple information with different relevance, in order to choose the most—suitable option. We designed a MCDA for new oral anticoagulants and warfarin integrating clinical events (benefits and risks) with drug cost to find the best oral anticoagulant from doctor perspective.

## Materials and methods

**Fig 1:**
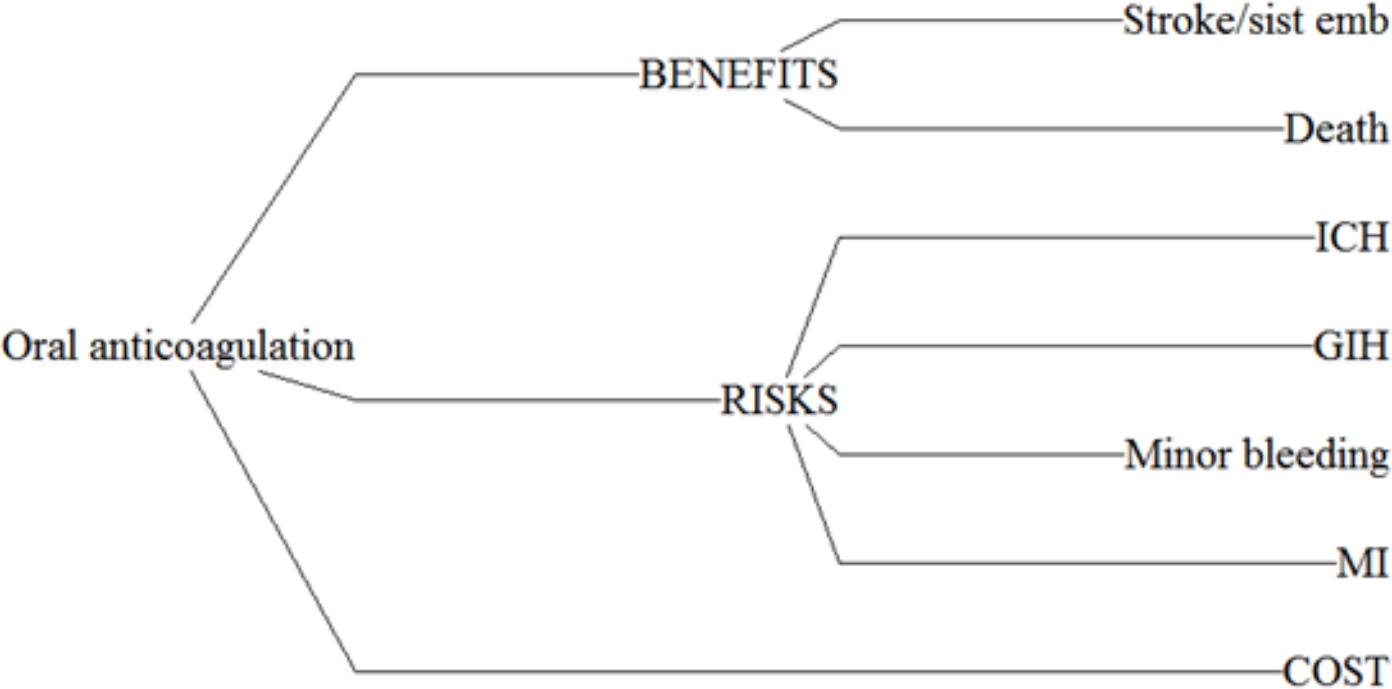
Benefits and risk included in the model.

To help formulate our decision context and define our value attributes, we used the guidelines of structured benefit-risk assessment by IMI-PROTECT (Pharmacoepidemiological Research on Outcomes of Therapeutics by European Consortium, [13] [14] and considerations of emerged good practice to perform MCDA by ISPOR [15] [16], we published the protocol found on dx.doi.org/10.17504/protocols.io.mwgc7bw. The assessment roadmap defines the following steps: 1 planning (focus on critical issues), 2 evidence gathering, and data preparation (data sources and extracts), 3 analyses (quantification of magnitudes), 4 explorations (robustness and sensitivity) and 5 conclusions (conclusion reached). 1. Planning: for the understanding of decision context, and as primary searching; we used the 2016 European guidelines and AHA/ASA guidelines for Atrial Fibrillation [17] [18], 2. For evidence gathering; we performed evidence synthesis of the existing literature, to explore the decision context in oral anticoagulation. The searching was made in MEDLINE, Cochrane and EMBASE of studies published in English, we used operators “AND” or “OR” or “NOT” for combining the following keywords: “atrial fibrillation”, “stroke”, “warfarin”, “Apixaban”, “Rivaroxaban”, “Dabigatrán”, we filtered by “systematic review”, “meta-analysis” “Clinical study”, “Clinical trial”. Database searches were made in May of 2017 without limitation of publication date. We excluded the following terms: “atrial ablation”, “ablation surgery” and “heparin”. 3. Analysis: for the selection of definitive criteria to include in our analysis, we took into account considerations made by Belton and Stewart for assessing the consequences of each option [19] (Fig 1). The following are these considerations: 3.1 *Value relevance*: the criteria was evaluated regarding the assumption that the data available in clinical trial and meta-analysis are relevant, 3.2 *Understandability*: we considered that there is a shared understanding and all criteria are clear in clinical terms; 3.3 *Measurability*: the availability of comparators between new oral anticoagulants versus warfarin, 3.4 *Non-redundancy*: we identified redundant criteria for the analysis, intracranial haemorrhage (ICH), gastrointestinal haemorrhage (GIH) are included in major bleeding so major bleeding could result in redundancy of this measure, 3.5 *Judgmental independence*: we considered that the majority of the criteria are preference independent, i.e. scores for one outcome are unaffected by scores for another outcome; 3.6 *Balancing completeness and conciseness*: we ensured that all critical aspects were captured and the level of detail was kept to a minimum, 3.7 *Operationality*: we developed a model to achieve a balance between completeness and conciseness.

### Scoring

The scoring of each criterion used in our model is summarized in Table 1, to construct the appropriate value scale for each criterion, we defined two reference points (0–100) and allocated numerical values to these points. The top of the scale was selected as the most preferred performance in which could realistically occur, and the bottom of the scale was selected as the least preferred option in which could realistically occur. We used a fixed scale to assess the performance of each treatment option for a given criteria. We took probabilities and cost from Harrington et al [20] cost-effectiveness analysis. These model inputs were entered in HiView software.

**Table 1:** Model inputs.

	Criterion	Most Preferred	Least Preferred	Warfarin	Apixaban	Dabigatrán	Rivaroxaban	Weight
**Benefits**	Risk of Stroke	0	1.10	1.10	0.88	0.91	1.10	90
	Risk of death	0	0.33	0.33	0.29	0.20	0.28	100
**Risks**	Risk of ICH	0	0.66	0.66	0.29	0.31	0.39	80
	Risk of GIH	0	1.56	0.89	0.58	1.54	1.56	60
	Risk of MB	0	17	11	9	13	17	40
	Risk of MI	0	0.80	0.68	0.49	0.80	0.71	60
**Cost**	Cost^¶^	0	327	$14	$327	$222	$222	40

Events rates taken from Harrington et al cost-effectiveness (20), ICH Intracranial hemorrhage, GIH Gastrointestinal hemorrhage, MB minor Bleeding, MI myocardial infarction

¶ annual cost.

### Weighing

We assigned weights for each criterion included in our analysis to reflect its relative importance. Table 1. We asked to experts their opinion about outcome importance of each criterion. We used a visual analogue scale and requested to set a point on it for each criterion using a scale from 0 to 100. A cardiologist, an internist and a vascular neurologist valued them regarding their preferences. Finally, we included weights as the average of physician’s responses. We identified the weights with consideration of clinical importance and without redundancy.

## Results

**Fig 2:**
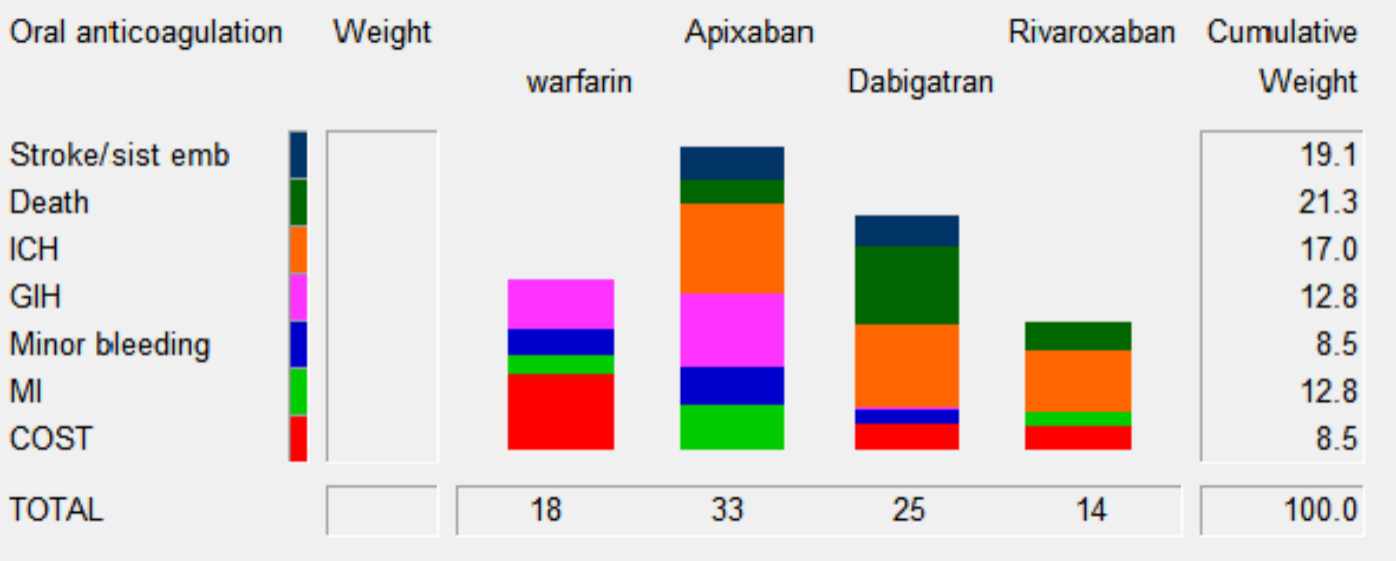
Criteria score by option.

The overall score for the multicriteria decision analysis was 33 for Apixaban, 25 for Dabigatrán, 18 for warfarin and 14 for Rivaroxaban Table 2. The highest score was for Apixaban and the lowest for warfarin, which indicates that Apixaban is the preferred treatment option. (Fig 2) below shows the contribution of benefits, risks, and costs to the overall score for each option. For our case, risks account the largest model weight followed by benefits and cost (51.1%, 40.4%, and 8.5% respectively). Apixaban accounted most for risk (it has less adverse events, which gives more model score), dabigatran 150mg accounted most for benefits (less stroke and death), warfarin accounted most for cost (is the cheaper option). (Fig 2) shows the criterion contribution for the total weights scores for each option, built up from the individual criterion. Death and intracranial haemorrhage are the highest value and cost is lowest value model weight. This figure also shows the different criteria contributions for each one, ICH is the criteria that most increases in NOACs but not in warfarin, but cost in warfarin adds relative weight, GIH adds weight to warfarin and Apixaban but more to the last. Death gives more value for Dabigatrán and minor bleeding for Rivaroxaban.

**Table 2:** Results.

	Weight	Warfarin	Apixaban	Dabigatran	Rivaroxaban	Relative Weight
**Benefits** **Risks** **Cost**	190 240 40	0 20 96	16 52 0	29 21 32	8 15 32	40.4 51.1 8.5
**Total**		**18**	**33**	**25**	**14**	**100**

### Sensitivity analysis

**Fig 3:**
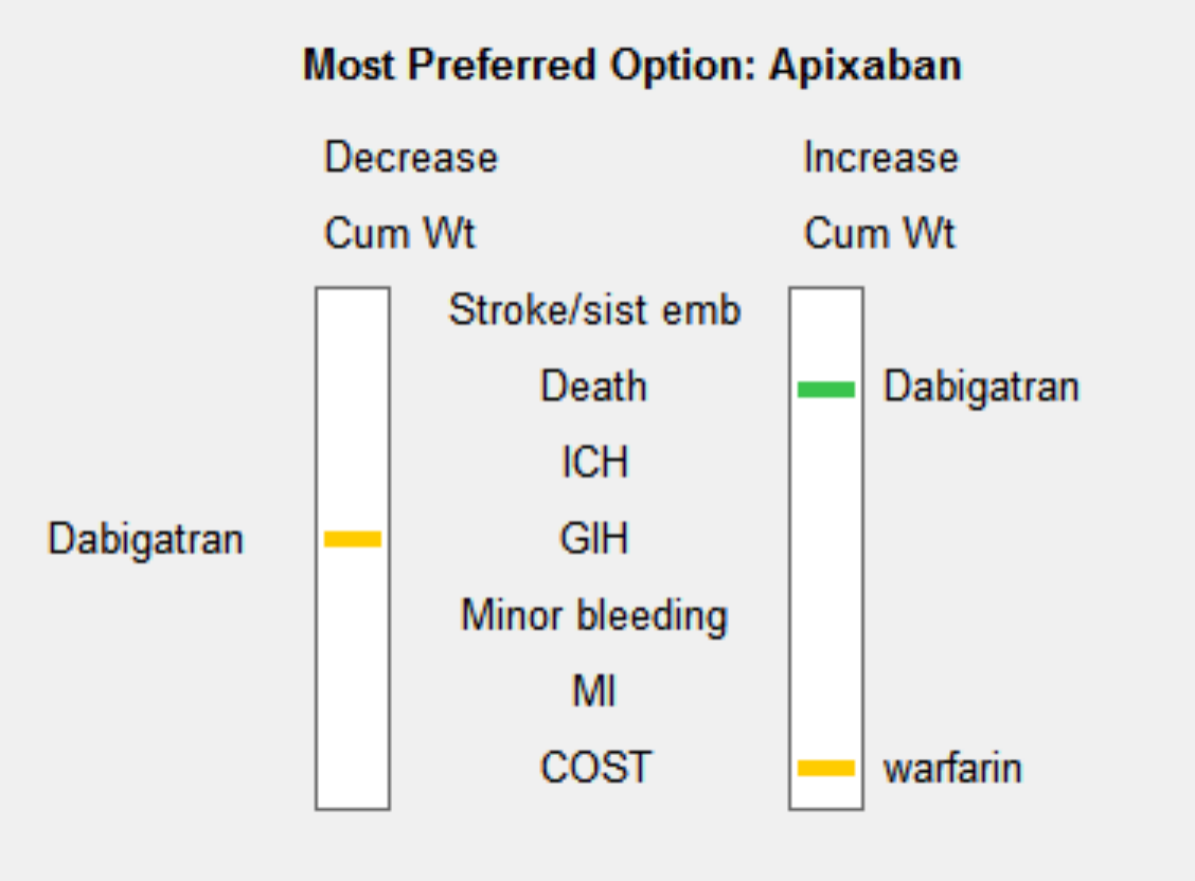
Sensitivity analysis down.

**Fig 4:**
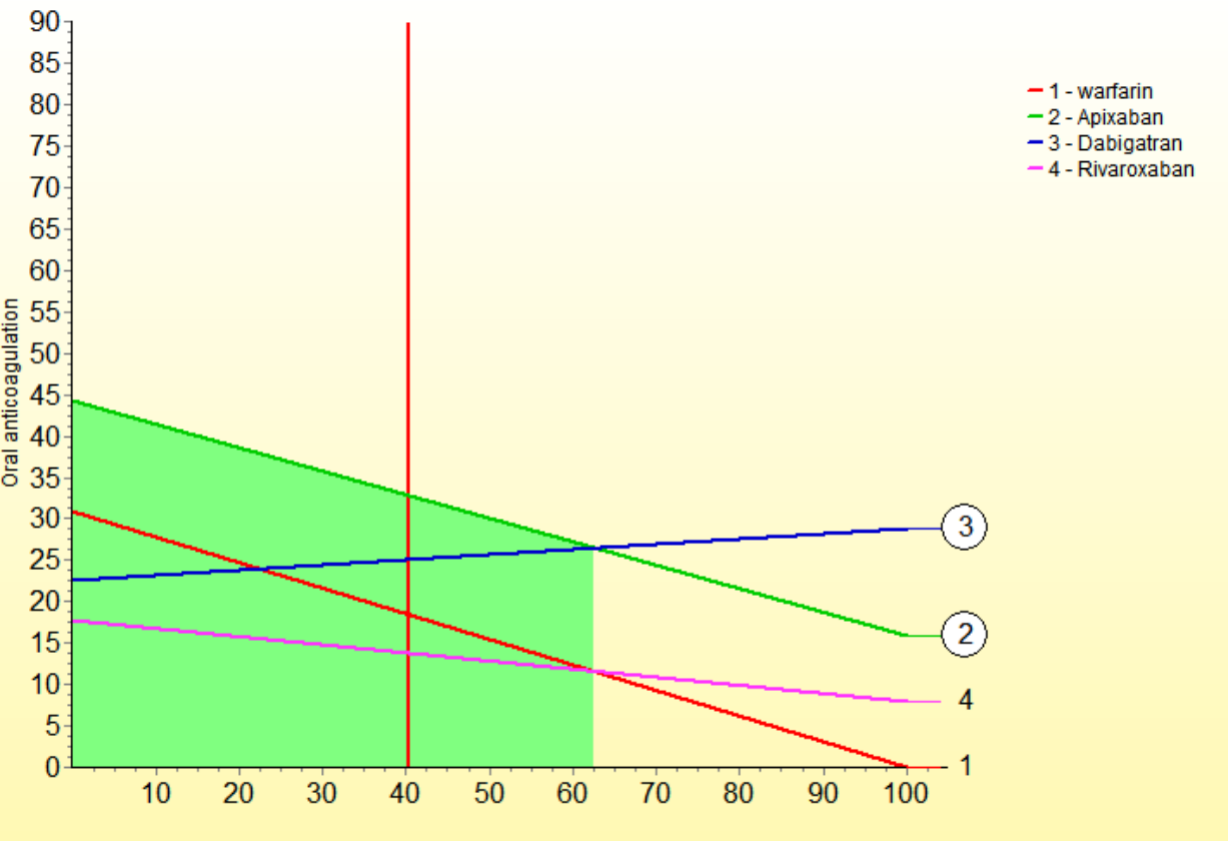
Sensitivity analysis up-down for benefits.

**Fig 5:**
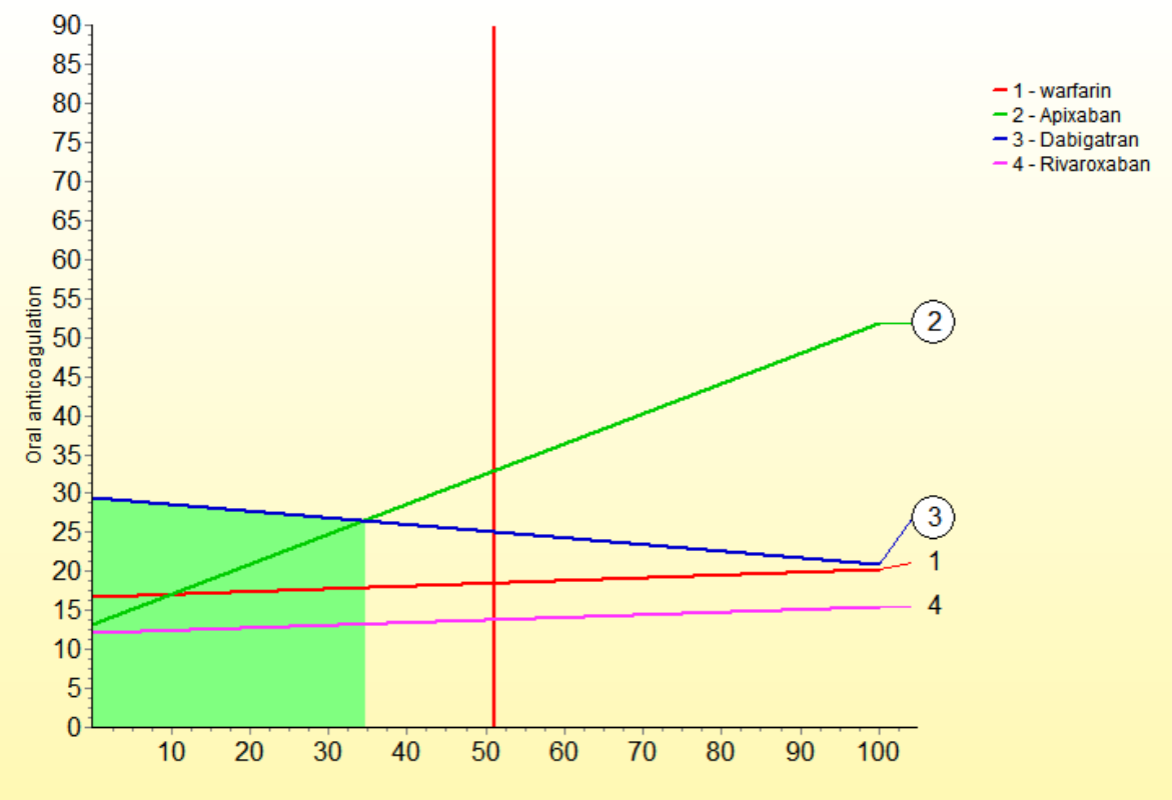
Sensitivity analysis up-down for risks.

**Fig 6:**
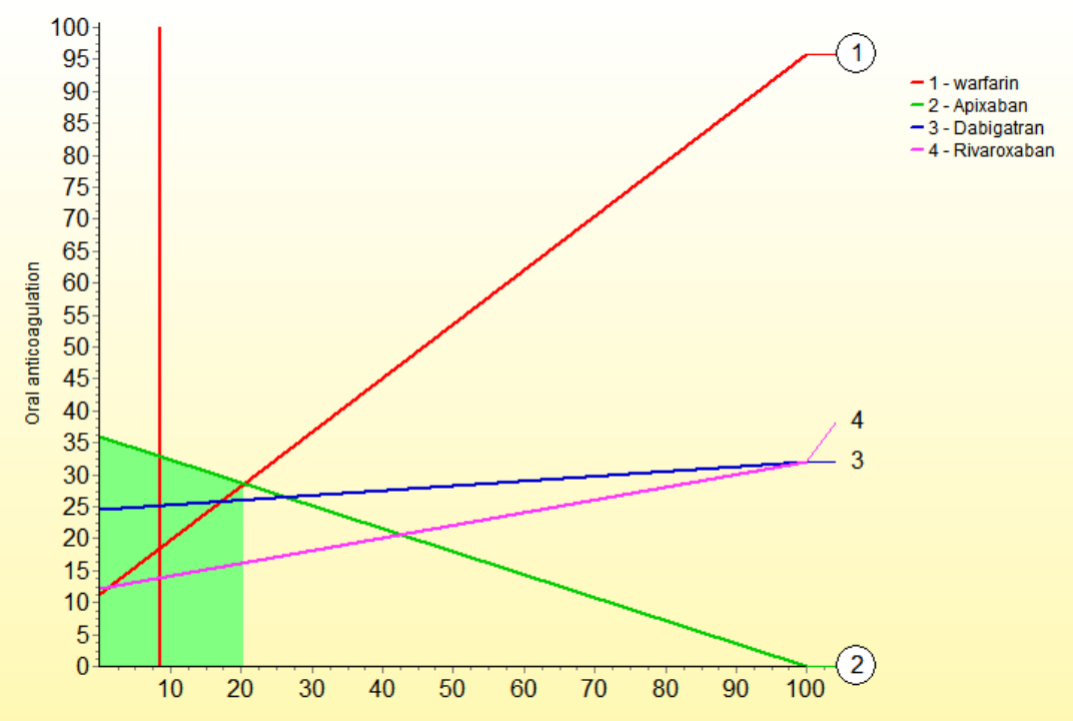
Sensitivity analysis up-down for costs.

We performed a series of sensitivity analysis to highlight areas in our model that might have a large influence on outcomes. The sensitivity down analysis (Fig 3) shows that dabigatran would be the most preferred option if death and GIH weights would change more than 15 and >5 to 15 points, and warfarin if cost weight would be >5 to 15 points more than current value. The sensitivity up analysis shows: for benefits (Fig 4), if its weight would rise to 60 points, dabigatran would be the preferred option; for risks (Fig 5), apixaban is the preferred option even if risks´ weight decrease to 35 points; for costs (Fig 6), if its weight rise to 20 points, warfarin would be the preferred option.

## Discussion

From this analysis apixaban is the preferred option followed by dabigatran, warfarin and rivaroxaban, this results from the inclusion of benefits, risk and drug´s cost. Apixaban is the preferred option due to better performance of risk and benefits compared to dabigatran; in this analysis, risks accounted 51.1% of model weight, benefits 40.4% and drug cost 8.5%. One reason for this result was the inclusion of more risk than benefits criteria (this because bleedings are relatively valuable and multiple) and the accountability of cost as only one criterion; thus, risks accounted more relative weight in this model.

The sensitivity analysis revealed that major changes must be done in order to choose another anticoagulant as the most preferred option: in the case that benefits weight rise more than 60% or if risks decrease up to 35% of model weight, then dabigatran would be the preferred option; and if costs rise to 20% of model weight, warfarin would be the preferred option. This shows scenarios on which criterion weight variation could affect the final decision, and confirms model robustness of this model. For criteria weight evaluation, we used an analogic linear scale to set a point on which doctors put which they best consider. Doctors gave less score (importance) to risks individually, and more for benefits that we think is realistic in clinical practice. Our results, of physician criterion weight are similar to Okamura et al study, on which they estimated the relative importance of nonfatal cardiovascular events using a discrete-choice experiment; therefore, it is unlikely that the most preferred option changes by doctor criterion weight. [21]

Our results differ from previous BRA by MCDA, Hsu et al, which showed that Dabigatran had the highest overall performance for general population (70 years old) and for primary and secondary prevention, but apixaban was the preferred option in patients with higher risk of stroke or in cases in which risk of extracranial bleeding is the main consideration. This analysis also differs from ours because weights were measured by a health utility method and also by the number of criteria included [12]. Tervonen et al, compared new oral anticoagulants for stroke prevention, and made two MCDA, one for consequences model and another for costs model; it revealed that dabigatran is the preferred option in both models [11]. This analysis differs ours because it included criterion as real-world evidence, interaction with food, availability of reversal agent and others that gave more criterion scores to dabigatran; costs model was evaluated as cost related with clinical events, which is useful to evaluate payer perspective (insurer). A potential criterion to include in future analyses is dementia as a consequence of AF in patients with cognitive impairment. Cacciatore et al, demonstrates that AF predicts dementia in elderly subjects, such information could be evaluated in relation to anticoagulant treatment that could be a new benefit of oral anticoagulants [22].

As limitations of this study we identified the use of information exclusively from pivotal clinical trials to scoring each criterion, we considered that inclusion of real world data can give a close overview, but this information is heterogeneous. Only few studies are published for two of the NOACs and can give advantage to these, in the case of including it. Other limitation is the generalization of endpoints; for the identification of sub-groups of patients that most can benefit of a particular anticoagulant, it is necessary to split out outcomes by group of patients or perform a personalized BRA to identify patients or environmental factors that modify absolute and/or relative benefits and harms [23]. Finally, the limitation of including costs as a criterion due to the controversy of the evaluation of opportunity cost in MCDA [16], but in this case doctors not buy drugs, so is only a consideration of cost importance for them [24].

One strength of this study is the inclusion of cost as a prescription factor by doctors; we wanted to evaluate in overall its influence on oral anticoagulants prescription. Considerations about anticoagulants costs are important in the perspective of the drug buyer (drugs excluded from a benefit plan in health systems) because out-of-pocket expenses affect medication adherence [25]. Other strength is the use of an integrate method to evaluated benefits-risks-costs in a quantitative way, which is useful to combine judgments and data in a transparent manner [26]; our analysis followed the steps proposed for MCDA good practices; our model is easy to understand for those that are no familiar with quantitative methods in BRA as doctors in clinical practice that could guide drug prescription.

The decision context for new oral anticoagulants from physician perspective reveals few impact of costs in the prescription, less than benefits or risks. Ghijben & col reported from patients preferences, that risk level of stroke and bleeding characteristics-as moderate to high- are the most important to them, and patients only would prefer warfarin if a NOAC cost $120AUD or more. This suggests that improving patient affordability may be important in the treatment rate of AF according to the authors [27]. This study shows the most value anticoagulant effect and their preference of cost, which can help doctors to reach a consensus with patients who pay their own medicines; for doing so, is necessary to know more thoroughly—patient preferences. A useful tool in Pharmacoepidemiology research is the conjoint analysis: it reveals preferences, satisfaction or utility of health care services, that represents a big step forward in terms of enhancing the benefits [28].

From this analysis apixaban is the preferred option followed by dabigatran, warfarin and rivaroxaban, this is due to less adverse events and similar benefits on each one. Costs, as doctor perspective, had little influence in the overall results but if cost was more valuated, apixaban would stop being the most preferred option. Special considerations should be made for each patient, and shared decisions may be necessary to improve medication adherence.
